# Supplementary material for: Predictors of metabolic syndrome among teachers in under-resourced schools in South Africa: Baseline findings from the KaziHealth workplace health intervention
Source: PLOS Glob Public Health. 2025 Jun 6;5(6):e0004681. doi: 10.1371/journal.pgph.0004681 (PMC12143548; doi:10.1371/journal.pgph.0004681)
Supplement: S1 Table — (DOCX) [file pgph.0004681.s001.docx]

**S1 Table: List of abbreviations**

| **Abbreviation** | **Definition** |
| --- | --- |
| AHA | American Heart Association |
| BMI | Body mass index |
| CI | Confidence interval |
| Crl | Credible interval |
| DBP | Diastolic blood pressure |
| EKNZ | Ethics Committee Northwest and Central Switzerland |
| ENMONZ | Euclidian norm minus 1, with negative values set to zero |
| ESS | Effective sample sizes |
| GEMS | Government Employees Medical Scheme |
| HbA1c | Glycated haemoglobin |
| HDL-C | High-density lipoprotein cholesterol |
| HICs | High-income countries |
| HIV | Human immunodeficiency virus |
| IDF | International Diabetes Federation |
| ISRCTN | International standard randomized controlled trial number |
| JIS | Joint Interim Statement |
| LMICs | Low- and middle-income countries |
| M | Mean |
| MetS | Metabolic syndrome |
| m*g* | Milligravity |
| MICE | Multiple imputation by chained equations |
| MRCSA | Medical Research Council of South Africa |
| MVPA | Moderate-to-vigorous intensity physical activity |
| n | Sample size / frequency |
| NCDs | Mon-communicable diseases |
| NHLBI | National Heart, Lung, and Blood Institute |
| NUTS | No-U-Turn Sampler |
| OR | Odds ratio |
| PMM | Predictive mean matching |
| PPC | Posterior predictive checks |
| $\hat{R}$ | Gelman-Rubin statistic |
| SBP | Systolic blood pressure |
| SES | Socioeconomic status |
| SD | Standard deviation |
| WHO | World Health Organization |
