# Supplementary figures and images for: Predictors of metabolic syndrome among teachers in under-resourced schools in South Africa: Baseline findings from the KaziHealth workplace health intervention
Source: PLOS Glob Public Health. 2025 Jun 6;5(6):e0004681. doi: 10.1371/journal.pgph.0004681 (PMC12143548; doi:10.1371/journal.pgph.0004681)

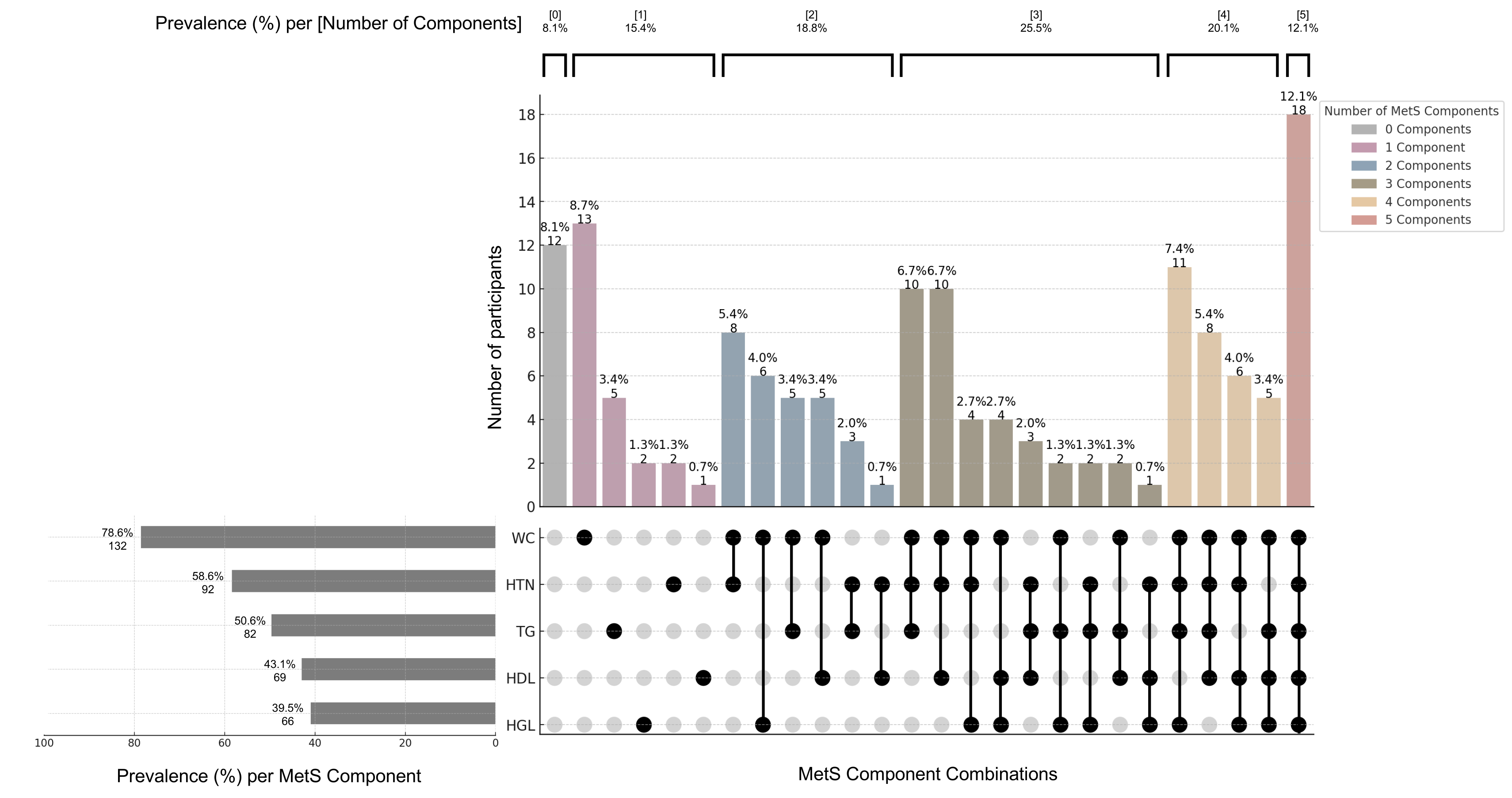

Supplement: S1 Fig — Legend for S1 Fig: MetS: Metabolic syndrome, WC: Central obesity (waist circumference with imputed values), HTN: Hypertension, TG: High triglyceride, HDL: Low high density lipoprotein cholesterol, HGL: Hyperglycaemia. (TIFF) [file pgph.0004681.s002.tiff]
