# Supplementary material for: Predictors of metabolic syndrome among teachers in under-resourced schools in South Africa: Baseline findings from the KaziHealth workplace health intervention
Source: PLOS Glob Public Health. 2025 Jun 6;5(6):e0004681. doi: 10.1371/journal.pgph.0004681 (PMC12143548; doi:10.1371/journal.pgph.0004681)
Supplement: Attachment — Submitted filename: mets_pa_ncdrisks_revision_01_pbp.docx [file pgph.0004681.s004.docx]

**Submission ID:**	PGPH-D-25-00167

**Title:**	Metabolic syndrome, physical activity, and modifiable risk factors of non-communicable diseases among teachers in under-resourced schools in South Africa: Baseline findings from the KaziHealth workplace health intervention

**Journal:**	PLOS Global Public Health

**Response to Reviewers**

24 March 2025

Dear Dr Buna Bhandari,

We refer to your e-mail dated 9 March 2025 and sincerely appreciate the opportunity to revise and resubmit our piece entitled “*Metabolic syndrome, physical activity, and modifiable risk factors of non-communicable diseases among teachers in under-resourced schools in South Africa: Baseline findings from the KaziHealth workplace health intervention*”, to PLOS Global Public Health. We are grateful for the insightful and constructive comments and suggestions provided by you and the two external reviewers, which have helped us strengthen the clarity, rigor, and overall quality of our work.

As per your request, we respond to each point raised below in blue text, with changes made to the manuscript highlighted using a yellow marker. We have further submitted an electronic copy of the latest version of the manuscript with track changes ('Revised Manuscript with Track Changes'), as well as a final clean version ('Manuscript').

We look forward to your further disposition and thank you again for all your efforts with our piece.

Kindest regards,

Nandi Joubert

(on behalf of all authors)

# Journal requests:

1. We have amended your Competing Interest statement to comply with journal style. We kindly ask that you double check the statement and let us know if anything is incorrect.

**Response:** Thank you. We have reviewed the updated ‘Competing Interest’ statement and confirm that it is correct.

2. We notice that your supplementary figure S1 is uploaded with the file type 'Figure'. Please amend the file type to 'Supporting Information'. Please ensure that each Supporting Information file has a legend listed in the manuscript after the references list.

**Response:** We have resubmitted the supplementary figure S1 as ‘Supporting Information’ and have ensured that it has a legend listed in the manuscript after the reference list.

3. In the online submission form, you indicated that “The data underlying this study will be made publicly available in Zenodo upon acceptance of the manuscript for publication to ensure alignment with the final published version. A DOI for the dataset will be provided in the final published article. For peer review purposes, the data are available from the corresponding author upon request.”.

All PLOS journals now require all data underlying the findings described in their manuscript to be freely available to other researchers, either

a. In a public repository,

b. Within the manuscript itself, or

c. Uploaded as supplementary information.

**Response:** Thank you, we have now uploaded the dataset supporting the conclusions of the paper on Zenodo and have updated the Data Availability Statement accordingly.

Data Availability Statement:

The dataset underlying this study is publicly available on Zenodo at [https://doi.org/10.5281/zenodo.15072311]. The dataset includes all variables necessary to replicate the study's findings and is shared under a Creative Commons Attribution 4.0 International (CC-BY 4.0) license. For any additional information regarding the dataset, please contact the corresponding author.

4. Please concisely present conclusion based on the study findings in brief.

**Response:** Thank you for your feedback. We have revised the conclusion to be more concise and focused on the key study findings.

Line 662-678:

Our study highlights a substantial burden of NCD risk among public primary school teachers in under-resourced schools in Gqeberha, South Africa. We found a high prevalence and severity of MetS, primarily driven by obesity, hypertension, and elevated triglycerides. Physical inactivity, a diet high in total fat, and lower SES were significant contributors to MetS risk. These findings underscore the urgent need for targeted public health interventions that not only promote healthier lifestyle behaviours but also address structural barriers to health equity. Limited healthcare awareness, nutritious food, and safe opportunities for physical activity in low-resourced communities exacerbates the risk of NCDs, highlighting the necessity for comprehensive, sustainable, and multi-level interventions.

# Reviewer 1:

The study subject and context are an important on-going area of research in chronic illnesses and their modifiable risk factors. This study represents the baseline to establish a reference point prior to implementing an educational/behavioural intervention programme. This welcomed with enthusiasm considering that findings thereafter would benefit a vast at-risk population. However, as much as this phase of the study represents the reference point to evaluate the outcome of the intervention, is very important that it is conceived in the highest standard and rigor of scientific principles for validity of the proof of concept underpinning the dynamics of the problem phenomenon for which the intervention has been designed. There are few observations made in this review to contribute significantly to strengthening the study argument and they are as follows:

1. The title “Metabolic syndrome, physical activity, and modifiable risk factors for non-communicable diseases among teachers in under-resourced schools in South Africa: Baseline findings from the KaziHealth workplace health intervention” would benefit greatly through modification because the present title has not placed the variables in the correct order of explanatory variables and outcome variable.

Explanatory variables should appear first because they explain the dynamics of the outcome variable. Where "Metabolic syndrome" is placed first appear to be an explanatory variable whereas it is a clinical outcome. Again, physical activity is a modifiable risk factor for non-communicable diseases and therefore should be remove. Suggested correction should be:

“Predictors of metabolic syndrome among teachers in under-resourced schools in South Africa: Baseline findings from the KaziHealth workplace health intervention”. With this title rendered this way, the physical activity, dietary concerns and substance use becomes embedded as the modifiable risk factors and their antecedent factors collectively covered by the word "predictors".

Justification for this revision is that Metabolic syndrome is the term generally used for describing cluster of non-communicable diseases that explain biochemical disfunction of metabolic pathways leading poor glucose control that trigger blood pressure control among others. The major modifiable risk factor for these diseases is usually a risk behaviour such as physical activity, tobacco-use and diet, therefore risk factors for non-communicable diseases would obviously be primarily behavioural and their antecedents. There is observed syntax issue with the original title. The “Baseline findings from the KaziHealth workplace health intervention” is actually redundant and makes the reading of the title unnecessarily long.

**Response:** Thank you for your suggestion regarding the title. We appreciate your feedback on ensuring the correct ordering of explanatory and outcome variables. In response, we have revised the title as follows: "Predictors of metabolic syndrome among teachers in under-resourced schools in South Africa: Baseline findings from the KaziHealth workplace health intervention." Please note that we have retained "Baseline findings from the KaziHealth workplace health intervention" to clarify that the study is cross-sectional in design and to highlight the broader intervention project it forms part of.

2. Unfortunately, the study has not been guided by sound public health principles to facilitate elucidating the dynamics of the problem phenomenon. The entire study is not guided by behaviour theoretical framework and therefore weak and unable to provide elucidation of the dynamics of the identified problem phenomenon. This study without any theoretical framework cannot hypothesize the dynamics of the study. Contemporary high-grade research is not conceived this way. Kindly consider the recommended revision.

**Response:** Thank you for highlighting the importance of a strong conceptual foundation in public health research. We appreciate your suggestion to incorporate a theoretical framework to better elucidate the dynamics of the problem phenomenon.

This study, however, follows a physiological and epidemiological approach, focusing on quantifying the prevalence and association of well-established risk factors (physical activity, diet, and substance use) with metabolic syndrome. These relationships are well-documented in the literature and are not the subject of theoretical debate. While we acknowledge that these lifestyle behaviours are influenced by broader social and environmental determinants, as outlined in models such as the Social-Ecological Model by Bronfenbrenner [1], our study does not aim to investigate the behavioural mechanisms driving these risk factors. Instead, we focus on their measurable impact on metabolic syndrome in this population.

That said, we recognise the value of behavioural frameworks in interpreting results, particularly in understanding barriers to behaviour change and contextualising our findings within broader public health principles. For the KaziHealth workplace health intervention results, we will take relevant frameworks into account when analysing and discussing our results. This will help situate our findings within existing behavioural and public health literature. We hope this clarification addresses your concern and appreciate your insights on strengthening the study’s conceptual rigour.

# Reviewer 2:

Thank you for a well written paper on an important topic. My comments are intended to strengthen the article, so please accept them in this spirit:

1. Covariates not considered: health insurance, mental health, disability - should be mentioned in 'limitation'.

**Response:** Thank you for your valuable feedback. We appreciate your suggestion to consider additional covariates in the study. Since all public school teachers in South Africa have access to health insurance through the Government Employees Medical Scheme (GEMS), we have made this point clear in the manuscript:

Line 263-265:

A referral for further medical screening was provided for any elevated metabolic markers identified. Public school teachers in South Africa have access to health insurance through the employer subsidy to the Government Employees Medical Scheme (GEMS), facilitating follow-up healthcare and further health assessments.

We further acknowledge that mental health and disability were not accounted for as covariates. In response, we have updated the limitations section to reflect these omissions.

Line 641-643:

Fourth, mental health and disability were not included as covariates, which may have influenced the observed associations as both covariates can impact metabolic health and physical activity levels.

2. Please explain how the reader should interpret the Odds Ratio, given your statement (line 619) re: cross-sectional study design.

**Response:** Thank you for your comment. We acknowledge that causal inference is not determined solely by study design but also by the underlying theoretical framework and control for confounding factors. We have revised the manuscript and limitation section to clarify that our findings should be interpreted with caution given the observational nature of the study.

Line 500-503:

Given the observational nature of the study, the results should be interpreted with caution. The estimated effect sizes indicate the strength of the relationship between metabolic syndrome and its predictors but do not necessarily imply causal effects.

Line 643-646:

Fifth, this study is observational and cross-sectional in nature, requiring caution in interpreting the results. While the associations observed provide valuable insights, causal effects cannot be directly inferred without accounting for confounding factors and theoretical assumptions.

3. The conclusion would benefit from recommendations for further research in the field.

**Response:** Thank you for your valuable suggestion. We appreciate your emphasis on the need for further research in this field. In response, we have added a paragraph towards the end of the discussion section outlining key areas for future research. We hope this addition provides a clearer direction for ongoing research in this area.

Line 608-626:

Future research should explore whether the high prevalence of MetS observed in this study reflects a broader national trend among teachers in South Africa. This could be achieved through cost-effective approaches such as secondary data analysis or targeted subnational studies. Further incorporating covariates such as mental health, which impacts metabolic health, physical activity levels, and overall wellbeing could provide a more comprehensive understanding of the determinants of metabolic syndrome and inform targeted interventions. Additionally, given the substantial burden of MetS in this population, research evaluating potential interventions would be essential to inform effective prevention strategies. Effective interventions should extend beyond individual-level lifestyle modifications to incorporate community-based, environmental, occupational, policy, and health system approaches. Such interventions should promote regular NCD screening and education strategies to raise awareness, facilitate early detection and treatment, and support individuals and communities to adopt and sustain healthier behaviours, especially around physical activity and diet. Prioritising teachers' health is not only essential for their wellbeing but also for improving educational outcomes and fostering healthy habits among the children they teach. This is especially critical in low-resourced settings, where teachers often serve as the primary stable role models. Failing to address the burden of NCDs and metabolic syndrome among teachers risks a cascading crisis with severe implications for both the education sector and future generations.

4. General comment: there were more European than African authors. The lead author is based at a European institution, while only one senior author is based at an African institution. This is against the spirit of global health from a decolonial perspective. How can this be addressed?

**Response:** Thank you for your thoughtful comment on authorship representation. We are committed to fostering equitable South-North research partnerships in our research group’s collaborations. Of the 16 authors, 7 are from South Africa, ensuring strong local expertise and leadership. The first author, though currently based at a Swiss institution, is South African with dual affiliations at the Swiss Tropical and Public Health Institute/University of Basel (currently pursuing a PhD fellowship with funding from Switzerland) and the Nelson Mandela University in South Africa. One of the three senior authors is South African and served as the Principal Investigator (PI) of the study, ensuring the study remains firmly rooted in the local context.

We acknowledge the importance of decolonial perspectives in global health and remain committed to collaborative, locally informed research that benefits South African public health. We continue to work towards a more equitable South-North partnership, decolonising our research approaches, and ensuring practices that value local knowledge while delivering meaningful benefits to the populations we engage with. We appreciate this discussion and the opportunity to clarify our approach. Thank you again for raising this important point and for advocating for greater equity in global health research. Your commitment to these discussions is invaluable, and we appreciate your efforts in pushing for more inclusive and just research practices.

**References**

1.	Bronfenbrenner U. Toward an experimental ecology of human development. American Psychologist. 1977;32: 513–531. doi:10.1037/0003-066X.32.7.513
